# Students in Philadelphia

**Published:** 1860-05

**Authors:** 


STUDENTS IN PHILADELPHIA.—The medical classes in this city, during the past session, were undoubtedly larger than in any other city in the world. The number was 1275, an increase of 112 over the session of 1858–59. The number of graduates was 381. Nearly twice as many students are at present in the city, pursuing their studies at the different private associations, as were here last spring.

